# Early warning for acute pyelonephritis with sepsis: risk prediction model for patients without renal medullary necrosis lesions

**DOI:** 10.3389/fmed.2026.1845085

**Published:** 2026-08-12

**Authors:** Bisheng Li, Shenglong Yu, Yihao Liao, Honggang Yuan

**Affiliations:** 1 Department of Urology, Affiliated Central People’s Hospital, China Three Gorges University, Yichang, Hubei, China; 2 Yichang Central People’s Hospital, Yichang, Hubei, China; 3 Department of Medical Oncology, Affiliated Second People’s Hospital, China Three Gorges University, Yichang, Hubei, China; 4 The Second People’s Hospital of Yichang, Yichang, Hubei, China

**Keywords:** acute pyelonephritis without renal medullary necrosis lesions, logistic regression, MIMIC database, risk prediction model, sepsis

## Abstract

**Background:**

Sepsis is one of the key factors leading to mortality in hospitalized patients. Acute pyelonephritis, as a common upper urinary tract infection, serves as a significant source of infection, with some patients progressing to sepsis, which markedly increases mortality risk. This study aimed to utilize a large-scale public intensive care database (MIMIC-IV) to construct and validate a model specifically designed for predicting sepsis risk in such patients, thereby providing clinicians with accurate and practical early warning tools.

**Methods:**

This retrospective cohort study employed data from the MIMIC-IV database version 3.1, enrolling 663 patients. Demographic characteristics, comorbidities (Charlson Index), and multiple vital signs and laboratory parameters within the first 24 h of hospital admission were collected. Patients were randomly divided into a model development set (465 cases) and an internal validation set (198 cases) at a 7:3 ratio. Univariate logistic regression, cross-validation LASSO regression, and multivariate logistic regression models were performed. Discrimination was assessed using the area under the receiver operating characteristic curve (AUC), while calibration and decision curve analysis (DCA) were employed to evaluate calibration accuracy and clinical Sepsis incidence rates utility.

**Results:**

The sepsis incidence rates in the development set and validation set were 12.90 and 12.63%, respectively. LASSO regression identified five predictive variables for the final model: Charlson Index, bicarbonate, calcium, mean corpuscular volume (MCV), and admission age. Multivariate analysis revealed that the Charlson index (OR = 1.10, 95% CI: 0.96–1.27), admission age (OR = 1.02, 95% CI: 1.00–1.04), and MCV (OR = 1.05, 95% CI: 1.00–1.10) were independent risk factors, while bicarbonate (OR = 0.90, 95% CI: 0.83–0.98) and calcium (OR = 0.27, 95% CI: 0.16–0.43) served as protective factors. The model demonstrated good apparent discrimination with AUCs of 0.820 (95% CI: 0.757–0.883) in the development set and 0.830 (95% CI, 0.737–0.924) in the validation set; internal bootstrap optimism correction yielded a bias-adjusted AUC of 0.805, confirming robustness against overfitting. Calibration was satisfactory, with decision curve analysis confirming net clinical benefit across threshold ranges of 1–80% (development) and 1–76% (validation). At the Youden-optimized cutoff of 0.194, the model achieved a sensitivity of 0.667 and specificity of 0.864 in the development set, and 0.600 and 0.803, respectively, in the validation set.

**Conclusion:**

This study successfully established a sepsis risk prediction model for acute pyelonephritis hospitalized patients without renal medullary necrosis lesions, based on five routine indicators. The model demonstrated excellent predictive performance and clinical utility in internal validation, providing a bedside quantitative tool for instantaneous risk stratification at hospital admission, facilitating early clinical vigilance and prioritized monitoring in this specific population.

## 1 Introduction

Sepsis is a clinical syndrome caused by dysregulated host response to infection, which can lead to life-threatening organ dysfunction and represents a core cause of mortality in hospitalized patients. Early identification and intervention are critical (1–3). Acute pyelonephritis, as a common upper urinary tract infection, is one of the significant sources of sepsis. Once sepsis develops secondary to acute pyelonephritis, it significantly increases treatment complexity and mortality rates (4, 5). However, in existing clinical prediction studies, acute pyelonephritis is often analyzed as a homogeneous disease entity, which actually encompasses a heterogeneous population ranging from simple renal parenchymal inflammation to complications such as renal papillary necrosis and renal abscess (6, 7). This broad definition may obscure critical pathophysiological differences across different pathological stages, leading to predictive models based on this population being influenced by confounding factors related to late-stage complications, thereby hindering accurate identification of true high-risk individuals during the early stages of infection (8, 9).

Therefore, focusing the study on the specific pathological stage of “acute pyelonephritis without renal medullary necrosis lesions” holds significant value. This sub-type refers to an acute inflammatory state where infection is confined to the renal parenchyma without causing ischemic renal papillary necrosis, representing a relatively homogeneous and reversible “window period” in disease progression (10–12). From a pathophysiological perspective, renal medullary necrosis is an ischemic rather than merely inflammatory event (13). Once established, it triggers a “necroinflammation” autoamplification loop that may render systemic inflammation irreversible, thereby obscuring early warning signals (14). Thus, focusing on the pre-necrosis stage isolates a true therapeutic window wherein host responses remain dynamic and potentially modifiable—making this subgroup uniquely suited for early prediction rather than an artificial subdivision. The clinical ICD-9 diagnostic code is 59010. During this phase, systemic inflammatory responses have been initiated but have not yet entered an uncontrollable vicious cycle due to local tissue necrosis. Developing predictive models for this population essentially involves identifying individuals at risk of dysregulation during the critical pre-sepsis period by utilizing routine indicators reflecting early inflammation, metabolic status, and compensatory mechanisms, thereby providing evidence for ultra-early clinical vigilance and risk-stratified management (15–17).

While the use of public databases like MIMIC for constructing clinical prediction models has become a trend, and numerous predictive tools targeting generalized sepsis populations exist, there remains a critical gap in sepsis risk prediction models specifically designed for acute pyelonephritis patients without renal medullary necrosis lesions—a clinically well-defined pathological subgroup. Existing models either suffer from accuracy limitations due to population heterogeneity or rely on biomarkers that are difficult to widely obtain, thereby restricting their clinical applicability.

Based on this, this study utilized the MIMIC-IV database to construct and validate a risk model specifically designed for predicting the progression of acute pyelonephritis patients without renal medullary necrosis lesions to sepsis. By precisely defining the pathological stage of the study population, this study aims to overcome heterogeneity interference associated with traditional definitions. Additionally, only routinely available clinical indicators measured within the first 24 h of admission—including age, comorbidity burden, and standard serum biomarkers—were included to develop a concise, practical, and easily interpretable quantitative tool. Crucially, the highest clinical value lies not in predicting a temporally remote event, but in quantifying, at the very first hospital encounter, the baseline physiological derangement that signals a trajectory toward subsequent diagnostic confirmation of sepsis. This model not only holds promise for enhancing early risk identification capabilities for this patient subgroup but also provides novel insights for infectious disease early warning research based on pathological stage stratification.

## 2 Methods

### 2.1 Study design and data sources

This study is a retrospective cohort study, with data sourced from the publicly available MIMIC-IV database (v3.1). The database contains de-identified clinical data of hospitalized patients from a large academic medical center in the United States between 2008 and 2022. The study utilized publicly available data and obtained an ethics review exemption.

This study was conducted and reported in accordance with the Transparent Reporting of a multivariable prediction model for Individual Prognosis Or Diagnosis (TRIPOD) statement. The completed TRIPOD checklist is provided as Supplementary Material (18).

### 2.2 Inclusion and exclusion criteria

This retrospective cohort study identified patients based on their final discharge diagnoses.

#### 2.2.1 Inclusion criteria

Patients aged ≥18 years with a primary discharge diagnosis of acute pyelonephritis without renal medullary necrosis (ICD-9 code 59010).

#### 2.2.2 Exclusion criteria

1. Discharge diagnosis of acute pyelonephritis with renal medullary necrosis (ICD-9 code 59011).
2. Sepsis onset occurred within the first 24 h of hospital admission. This ensured that all predictor variables were measured before the outcome, allowing for true prediction.
3. Missing values exceeding 20% for key predictor variables.

**Figure 1:**
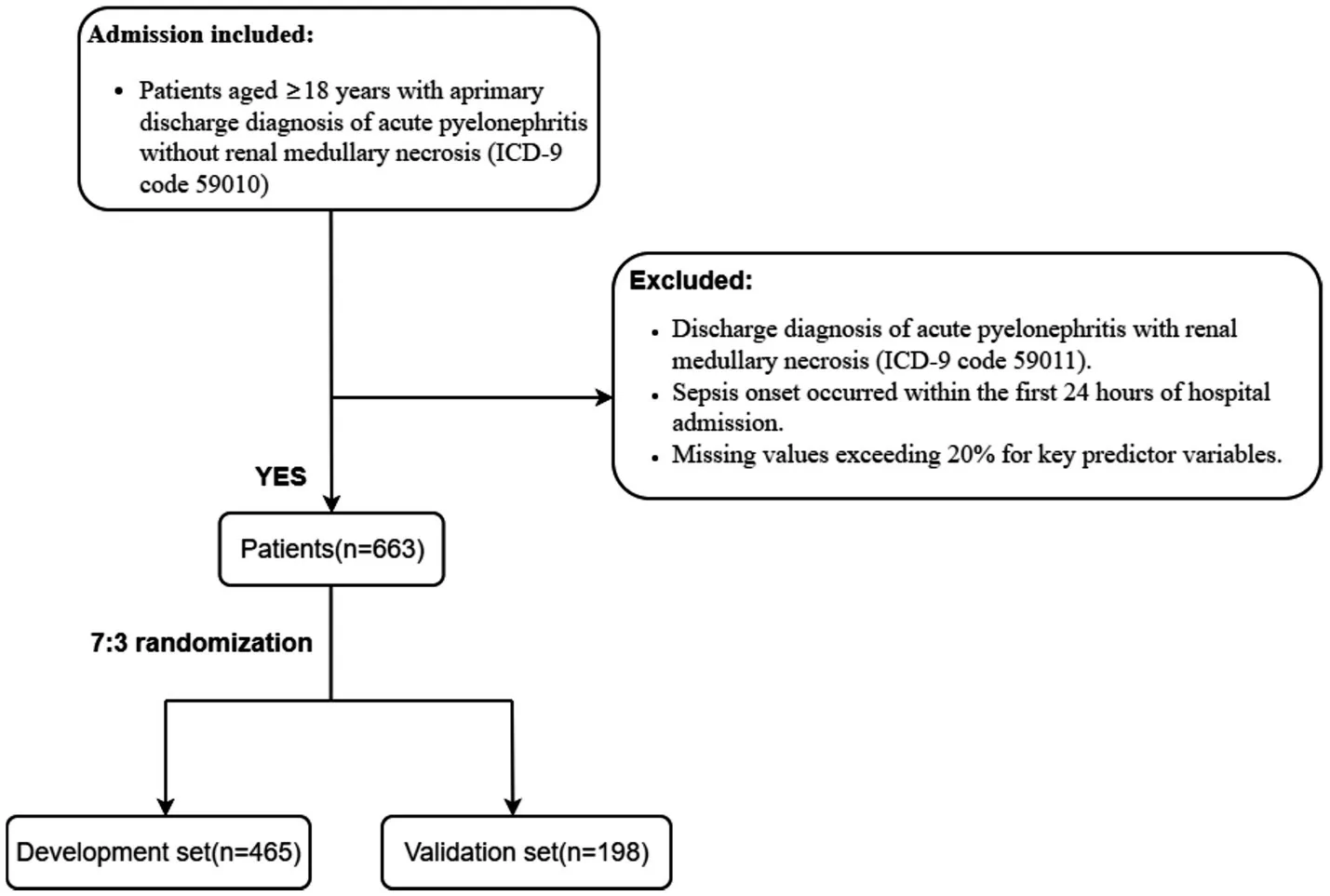
Flowchart of study subject screening process.

This design yielded a cohort in which all baseline variables were extracted from the first 24 h, and sepsis could only occur after that landmark, or not at all. We additionally verified, using individual measurement timestamps available in the MIMIC-IV database, that for all retained patients the first available measurement of each predictor preceded the respective sepsis3_onset_time, thereby confirming temporal precedence at the patient level (Figure 1).

### 2.3 Variable definition and data extraction

*Outcome variable*: Sepsis occurrence was defined according to Sepsis-3.0 criteria, identified via the sepsis3_inclusion_switch flag in the database. To ensure temporal validity, we defined a landmark at 24 h after hospital admission. Patients whose sepsis3_onset_time occurred before this landmark were excluded. Thus, the outcome represents sepsis diagnosed after the 24-h landmark, ensuring that all predictors preceded the outcome.

*Predictive variables*: Baseline demographic data, comorbidities, and routine laboratory parameters were extracted from the first 24 h of hospital admission. For each predictor, the first available measurement within this window was used. A total of 37 candidate variables were collected:

1. *Demographics and baseline*: Age, gender.
2. *Comorbidities*: Charlson Index, cerebrovascular diseases, chronic pulmonary diseases, congestive heart failure, dementia, diabetes, hypertension, malignant tumors, metastatic solid tumors, mild liver disease, myocardial infarction, paraplegia, peptic ulcer disease, peripheral vascular diseases, renal diseases, rheumatic diseases, severe liver disease.
3. *Laboratory parameters*: Including internal environment (bicarbonate, anion gap, sodium, potassium, chloride, calcium, magnesium, phosphate), renal function (creatinine), perfusion (lactate), hematologic system (hemoglobin, platelets, hematocrit, red blood cell distribution width, MCV, mean corpuscular hemoglobin content, mean corpuscular hemoglobin concentration).

### 2.4 Data processing and statistical analysis

Analysis was performed using R software (version 4.5.0).

1 *Data preprocessing*: Categorical variables were converted to factors, and continuous variables were transformed into numerical types. All predictor variables were extracted within the predefined 24-h window (admission to landmark). Variables with more than 20% missing values were excluded. For the remaining variables with missing data, multiple imputation using chained equations (MICE) was performed with 5 imputations and 5 iterations, incorporating all candidate predictors and the outcome variable in the imputation model to minimize bias. Clinical outliers were subjected to tail-trimming treatment.
2 *Dataset partitioning*: The dataset was randomly divided into a development set (465 cases) and a validation set (198 cases) using the caret package in a 7:3 ratio to ensure balanced outcome distribution.
3 Feature screening and model construction:

*Univariate analysis*: Univariate logistic regression was performed for each candidate variable in the development cohort. This step was descriptive in nature, intended to provide an overview of the associations between individual predictors and the outcome, and to inform the subsequent regularization step. Variables with *p* < 0.05 were considered for entry into the LASSO regression; however, this threshold was not used as a standalone filtering criterion for variable selection.

*LASSO regression for variable selection*: To select a parsimonious set of predictors while mitigating overfitting and multicollinearity, LASSO regression with 10-fold cross-validation was performed using the glmnet package. The penalty parameter *λ* was selected using the 1-standard error (1-SE) rule, yielding λ₁ₛₑ = 0.051043. This step served as the primary variable selection mechanism, yielding five non-zero coefficient predictors.

*Multivariable logistic regression for final model construction*: The five predictors selected by LASSO were then entered into a standard multivariable binary logistic regression model. This step was performed to construct the final interpretable prediction model, allowing for the calculation of adjusted odds ratios (OR) and 95% confidence intervals (CI) for each predictor, and to generate a clinically applicable nomogram. No further variable selection was performed at this stage; the final model coefficients were estimated using the fixed set of LASSO-selected predictors.

4 Model performance evaluation:

*Discrimination*: Calculate the AUC between the development set and the validation set.

*Calibration accuracy*: Plot calibration curves and perform the Hosmer-Lemeshow test.

*Clinical applicability*: Conduct decision curve analysis to evaluate clinical net benefit at different threshold probabilities.

5 *Visualization*: Plot bar charts using the “rms” package based on the final model.

## 3 Results

### 3.1 Study population characteristics

From an initial candidate pool of 689 patients, after applying the 24-h landmark restriction and verifying temporal precedence via individual timestamps, 26 patients were excluded, resulting in a final analytic cohort of 663 patients with acute pyelonephritis without renal medullary necrosis lesions. The overall sepsis incidence rate was 12.82% (85/663). The development set and validation set showed no statistically significant differences in age, gender, Charlson Index, key laboratory parameters, or sepsis incidence rate (all *p* > 0.05), indicating balanced grouping. The incidence rates were 12.90% (60/465) in the development set and 12.63% (25/198) in the validation set.

### 3.2 Selection of predictive factors and model

**Figure 2:**
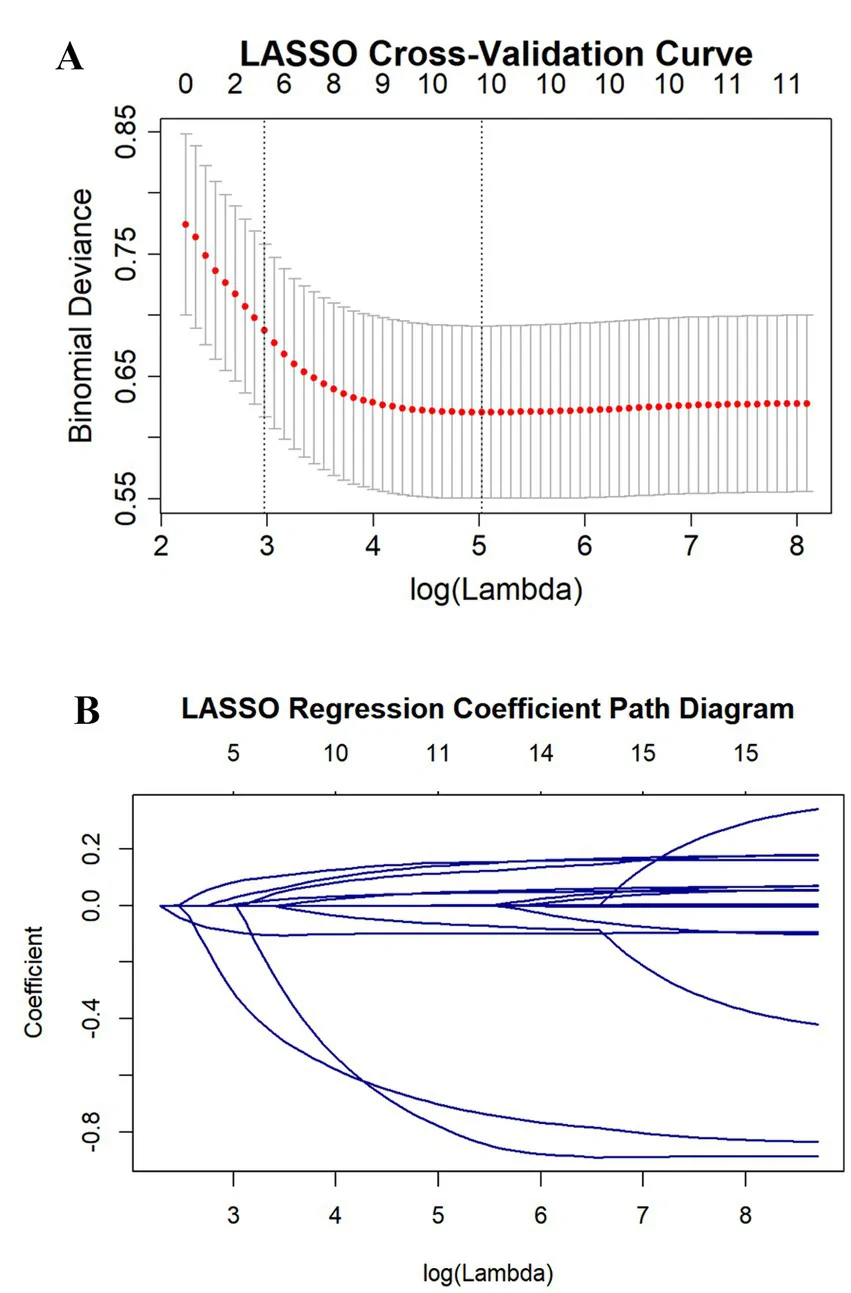
Variable selection using LASSO regression with 10-fold cross-validation. **(A)** Cross-validation curve for the penalty parameter *λ*. The left vertical dashed line indicates *λ_min*, and the right vertical dashed line indicates *λ₁ₛₑ* (0.051043), selected for the parsimonious model. **(B)** LASSO coefficient profiles of the 11 significant variables against log(*λ*). Only 5 variables retained non-zero coefficients at the selected *λ₁ₛₑ*: Charlson index, bicarbonate, calcium, MCV, and admission age.

Univariate associations for all candidate variables are presented in Table 1. LASSO regression (*λ*₁ₛₑ = 0.051043) selected 5 non-zero coefficient variables (Figure 2): Charlson index, bicarbonate, calcium, MCV, and admission age. Multivariable logistic regression demonstrated that bicarbonate (OR = 0.90, 95%CI: 0.83–0.98) and calcium (OR = 0.27, 95%CI: 0.16–0.43) were significant protective factors, while MCV (OR = 1.05, 95%CI: 1.00–1.10) was a significant risk factor. Charlson index (OR = 1.10, 95%CI: 0.96–1.27) and admission age (OR = 1.02, 95%CI: 1.00–1.04) showed non-significant associations but were retained in the model based on LASSO selection. Multicollinearity was assessed using the variance inflation factor (VIF) for the five predictors incorporated in the final multivariable model. The VIF estimates ranged from 1.030 to 2.152, all substantially below the conventional threshold of 10, indicating that multicollinearity was not a significant concern.

**Table 1:** Univariate and multivariable associations of predictive factors acute pyelonephritis without lesion of renal medullary necrosis.

Variable	Univariate		Multivariate		VIF
	OR (95% CI)	*p* value	OR (95% CI)	*p* value	
admission_age	1.03 (1.01–1.04)	<0.001	1.02 (1.00–1.04)	0.088	2.152
aids	0.00 (0.00-Inf)	0.986			
aniongap	1.13 (1.04–1.22)	0.003			
bicarbonate	0.82 (0.76–0.88)	<0.001	0.90 (0.83–0.98)	0.019	1.090
calcium	0.26 (0.17–0.39)	<0.001	0.27 (0.16–0.43)	<0.001	1.188
cerebrovascular_disease	3.54 (1.03–12.16)	0.044			
charlson	1.18 (1.08–1.28)	<0.001	1.10 (0.96–1.27)	0.174	2.095
chloride	1.09 (1.03–1.16)	0.004			
chronic_pulmonary_disease	1.84 (0.97–3.51)	0.062			
congestive_heart_failure	4.24 (2.15–8.34)	<0.001			
creatinine	1.25 (1.04–1.49)	0.016			
dementia	1.96 (0.40–9.67)	0.408			
diabetes	1.58 (0.89–2.81)	0.12			
first_hosp_stay_switch	1.24 (0.72–2.13)	0.441			
gender	0.93 (0.48–1.79)	0.823			
hematocrit	0.94 (0.89–1.00)	0.053			
hemoglobin	0.81 (0.68–0.96)	0.016			
hypertension	1.26 (0.72–2.20)	0.412			
magnesium	0.17 (0.07–0.43)	<0.001			
malignant_cancer	0.92 (0.27–3.16)	0.89			
mch	0.88 (0.73–1.06)	0.172			
mchc	0.88 (0.74–1.06)	0.193			
mcv	1.08 (1.04–1.12)	<0.001	1.05 (1.00–1.10)	0.03	1.030
metastatic_solid_tumor	1.36 (0.29–6.37)	0.695			
mild_liver_disease	3.58 (1.54–8.35)	0.003			
myocardial_infarct	4.39 (1.53–12.56)	0.006			
paraplegia	1.37 (0.38–4.88)	0.628			
peptic_ulcer_disease	2.27 (0.23–22.20)	0.481			
peripheral_vascular_disease	2.36 (0.83–6.76)	0.109			
phosphate	1.14 (0.92–1.42)	0.242			
platelet	0.99 (0.99–1.00)	<0.001			
potassium	1.08 (0.64–1.83)	0.765			
rdw	1.12 (0.98–1.28)	0.086			
renal_disease	1.38 (0.72–2.65)	0.327			
rheumatic_disease	1.07 (0.31–3.73)	0.916			
severe_liver_disease	6.85 (0.42–110.96)	0.176			
smoker	26.61 (5.39–131.47)	<0.001			
sodium	1.02 (0.94–1.10)	0.66			

mch, Mean corpuscular hemoglobin; mchc, Mean corpuscular hemoglobin concentration; mcv, Mean corpuscular volume; rdw, Red blood cell distribution width. CI, Confidence interval; OR, Odds ratio.

### 3.3 Model performance

#### 3.3.1 Discrimination ability

**Figure 3:**
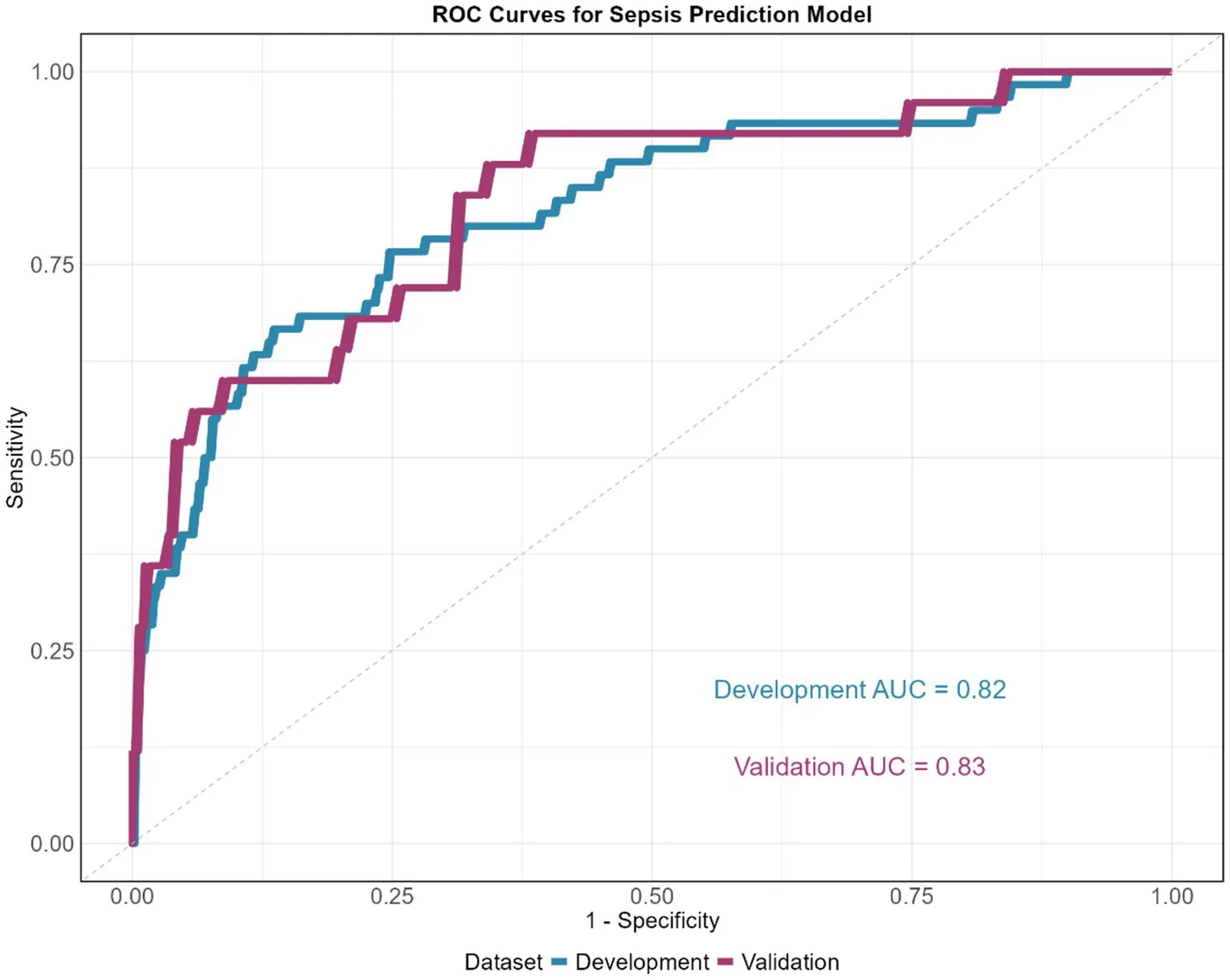
Receiver operating characteristic (ROC) curves of the prediction model for sepsis in acute pyelonephritis without renal medullary necrosis. The area under the curve (AUC) was 0.820 (95% CI, 0.757–0.883) in the development cohort and 0.830 (95% CI, 0.737–0.924) in the internal validation cohort, indicating good discriminative ability. AUC comparisons were performed using the DeLong method.

The model demonstrated good and stable discrimination capability with an AUC of 0.820 (95% CI: 0.757–0.883) on the development set and 0.830 (95% CI: 0.737–0.924) on the validation set (Figure 3).

#### 3.3.2 Internal validation was performed using 500 bootstrap resamples to estimate and correct for optimism

The apparent performance of the model on the development set (using the Youden-optimized cutoff of 0.194) was AUC = 0.820, accuracy = 0.839, sensitivity = 0.667, and specificity = 0.864. After optimism correction, the bias-adjusted estimates were AUC = 0.805, accuracy = 0.833, sensitivity = 0.646, and specificity = 0.862. The optimism values were 0.015 for AUC, 0.006 for accuracy, 0.021 for sensitivity, and 0.003 for specificity, indicating that overfitting was modest and adequately accounted for.

#### 3.3.3 Calibration

**Figure 4:**
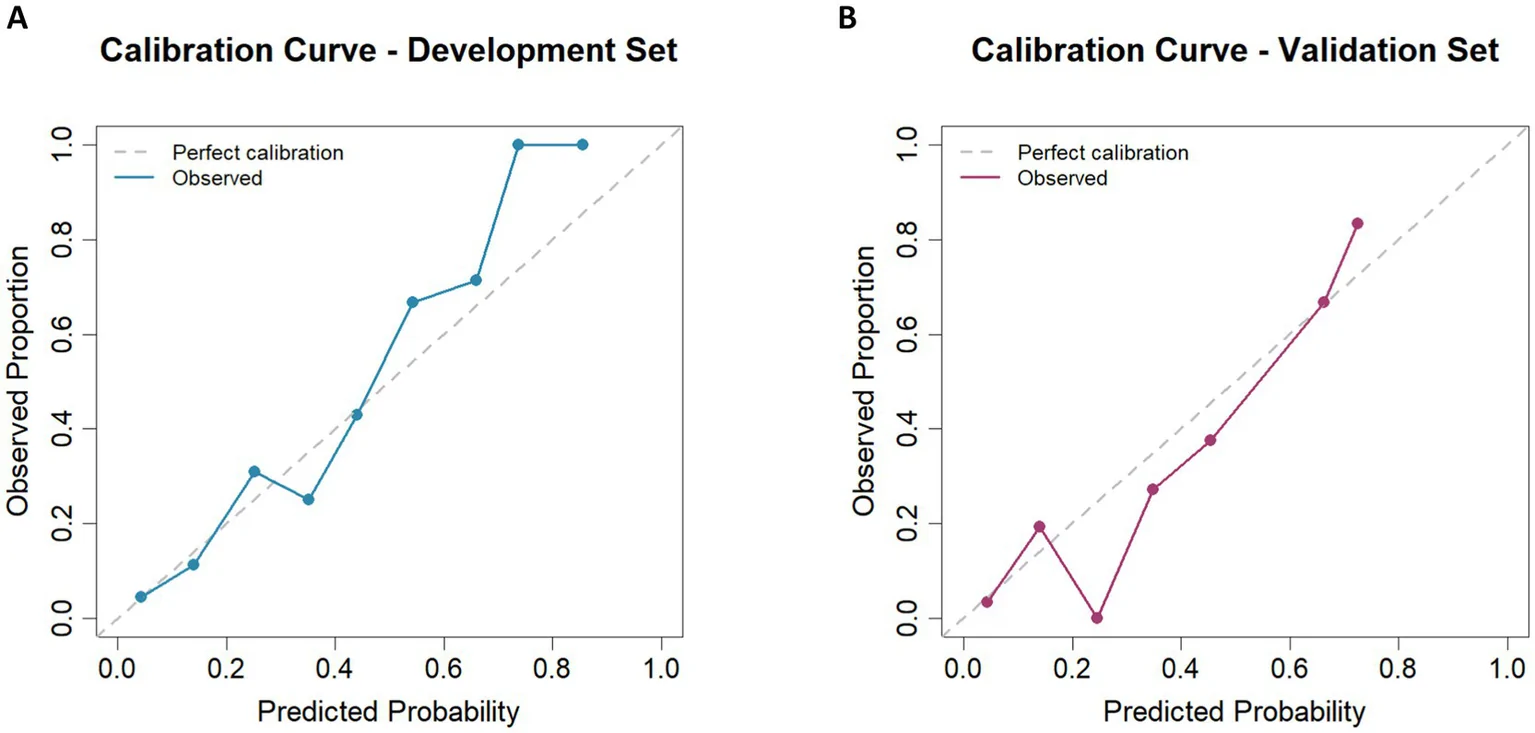
Calibration plots for the prediction model in the **(A)** Development and **(B)** Validation cohorts. The x-axis represents the predicted probability of sepsis, and the y-axis represents the observed proportion. The dashed diagonal line indicates perfect calibration. The smooth loess curve (red dashed line) shows the observed-to-predicted relationship. Hosmer-Lemeshow test *p*-values were 0.976 and 0.079 for the development and validation sets, respectively, supporting good calibration.

The calibration plots for both the development and validation sets are presented in Figure 4. In the development cohort, the calibration curve closely followed the ideal 45-degree diagonal line, indicating good agreement between predicted probabilities and observed sepsis rates. The Hosmer-Lemeshow test yielded a non-significant *p*-value of 0.976, supporting adequate model fit. In the validation cohort, the calibration curve also demonstrated satisfactory alignment with the diagonal, with a Hosmer-Lemeshow *p*-value of 0.079, suggesting no significant miscalibration despite the smaller sample size. These results confirm that the model’s predicted risks are well-calibrated and reliable for clinical application (Figure 4).

#### 3.3.4 Clinical applicability

**Figure 5:**
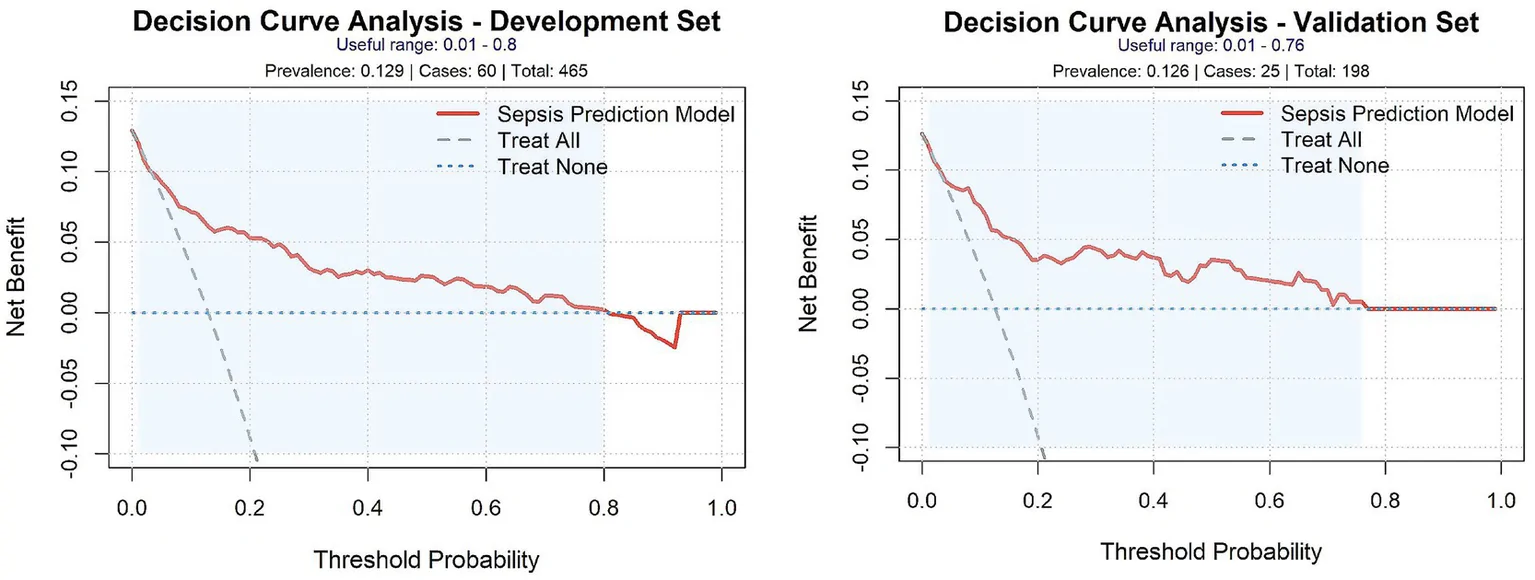
Decision curve analysis (DCA) for the prediction model in the development and validation sets. The *y*-axis indicates net benefit. The model (red line) showed superior net benefit compared to the “treat all” (gray dashed line) and “treat none” (blue dotted line) strategies across threshold probability ranges of 1 to 80% in the development set and 1 to 76% in the validation set, supporting its clinical utility.

Decision curve analysis demonstrated that the clinical net benefit of using this model was higher than that of the “treat all” or “treat none” strategies across a threshold probability range of 1 to 80% in the development set and 1 to 76% in the validation set (Figure 5), supporting its clinical utility for decision-making (Figure 5).

#### 3.3.5 Forecast line chart

**Figure 6:**
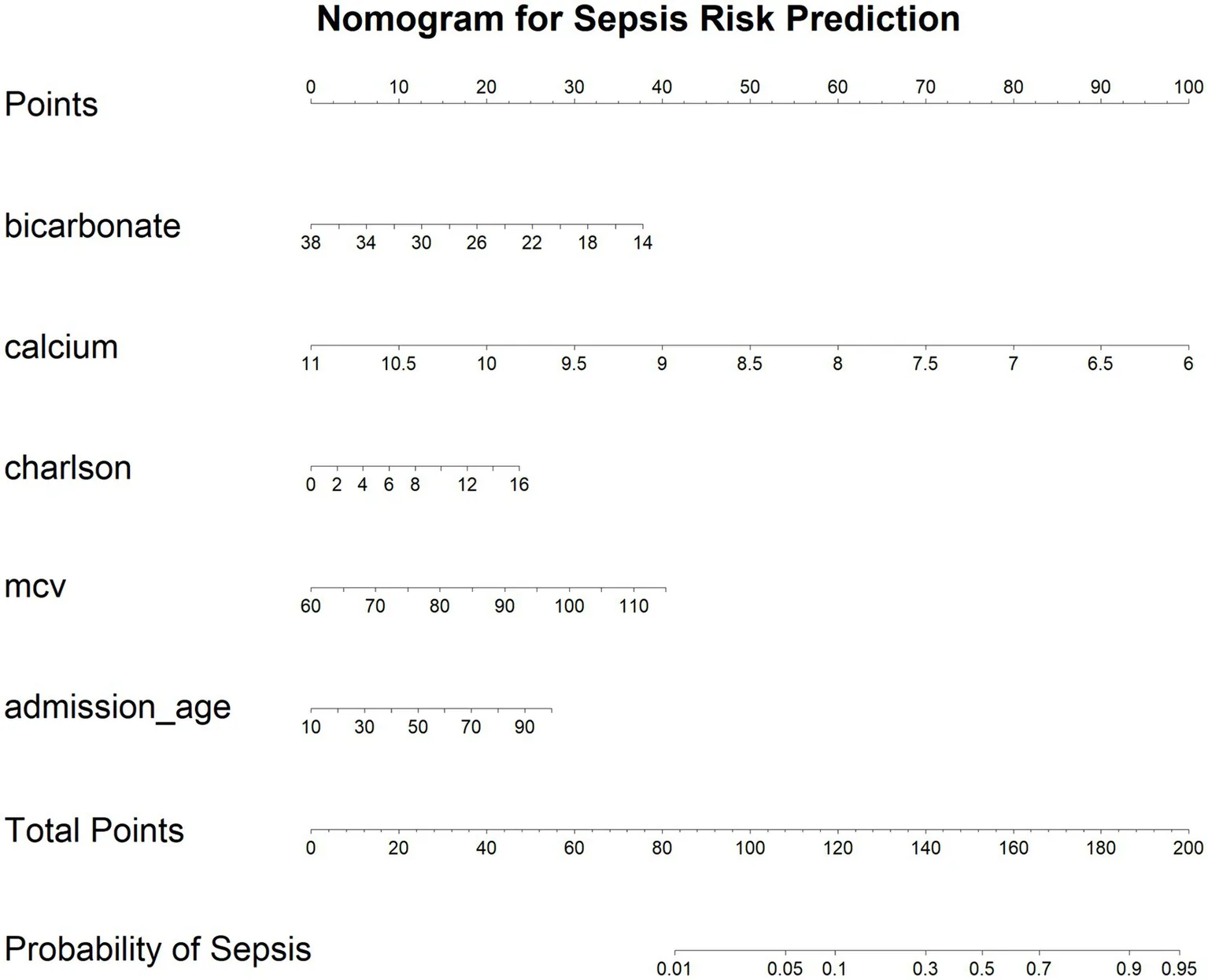
Nomogram for predicting the individual probability of sepsis in patients with acute pyelonephritis without renal medullary necrosis. The five predictor variables are: Charlson index (comorbidity burden), bicarbonate (mEq/L), calcium (mmol/L), mean corpuscular volume (fL), and admission age (years). To use the nomogram, locate each predictor on its respective scale, draw a vertical line to the “Points” axis to assign points, and sum the points for all variables to obtain a total score. A vertical line from the total score to the bottom “Probability of Sepsis” axis directly estimates the patient’s predicted risk (the nomogram was developed based on the final multivariable logistic model).

Based on the visualization of the final model, a nomogram was generated by summing the scores of the five indicators, which directly corresponds to the individual probability of sepsis occurrence, facilitating rapid clinical application (Figure 6).

## 4 Discussion

This study developed and internally validated a five-variable prediction model for sepsis in patients with acute pyelonephritis without renal medullary necrosis. By focusing on this reversible pre-necrosis stage, we reduced heterogeneity common in mixed cohorts. The model showed robust discrimination (AUC 0.820 development, 0.830 validation) and satisfactory calibration, with net clinical benefit across 1–80% (development) and 1–76% (validation) thresholds. All predictors are routine admission parameters, enabling bedside use without expensive biomarkers. The statistical methodology adopted rigorous variable selection via LASSO regression with 10-fold cross-validation, and internal validation through 500 bootstrap resamples yielded an optimism-corrected AUC of 0.805, with minimal optimism values for accuracy (0.006), sensitivity (0.021), and specificity (0.003), confirming that overfitting was modest and the model’s performance is reliably generalizable. To further assess the stability of the final model, we examined multicollinearity among the five selected predictors using the variance inflation factor (VIF). The VIF values ranged from 1.030 to 2.152, well below the conventional threshold of 10, indicating negligible collinearity. This result ensures that each predictor contributes largely independent information, supporting the reliability of the estimated odds ratios and reinforcing the interpretability of the nomogram for bedside application.

The predictive relevance of these variables is supported by prior evidence directly linking them to sepsis risk or adverse outcomes in infected patients. The Charlson comorbidity index captures baseline physiological vulnerability and has been consistently identified as an independent predictor of sepsis development and mortality (19, 20). Advanced age is a well-established risk factor for sepsis, with immunosenescence, diminished physiological reserve, and higher comorbidity burden contributing to increased susceptibility and poorer outcomes (21). Serum bicarbonate exhibits a U-shaped relationship with mortality, with both extremes conferring higher risk (22). Hypocalcaemia has emerged as a strong prognostic indicator in sepsis, independently predicting poor outcomes (23, 24). Mean corpuscular volume, though traditionally overlooked, has recently been correlated with sepsis mortality, possibly reflecting chronic inflammation or impaired hematopoietic reserve (25). These indicators, all obtainable from routine admission panels, collectively provide a practical and evidence-based foundation for early risk stratification in this specific subgroup. Several limitations warrant consideration. The sepsis3_inclusion_switch flag in the database is retrospectively derived from the entire hospitalization, which may introduce temporal ambiguity. To counter this, our study protocol strictly aligned with the prespecified inclusion/exclusion criteria: we excluded patients with sepsis onset within the first 24 h, and extracted all predictors exclusively from measurements obtained during that same initial window. Consequently, the outcome was defined as sepsis diagnosed after the 24-h landmark, ensuring that all predictors preceded the outcome and eliminating label leakage. This design positions our model specifically for early risk stratification at admission, not for predicting events within the first 24 h or capturing subsequent dynamic changes. Thus, the model should be used as a quantitative aid to complement, not supplant, clinical judgment, offering objective support for early vigilance and prioritized monitoring in this defined population.

Beyond this, the retrospective single-center design introduces potential selection bias and limits generalizability; prospective multi-center external validation is essential. Statistically, the relatively modest sample size (663 patients) and low event rate (12.82%)—yielding approximately 12 events per variable in the final model (85 events for 7 estimated parameters including intercept)—are adequate but limited, raising the possibility of overfitting despite bootstrap optimism correction (AUC optimism = 0.015). The LASSO regression, while effective for variable shrinkage, may have excluded clinically relevant predictors with weaker marginal effects. Missing data were handled via multiple imputation, but the missing-at-random assumption remains untestable and may introduce bias if missingness is informative. All predictors were measured at a single time point; static measurements cannot capture evolving physiological changes, and we did not adjust for time-dependent interventions (e.g., fluid resuscitation, vasopressors) that could modify the natural course.

The outcome definition relied on an administrative sepsis flag, which may be subject to misclassification, and case identification via ICD-9 code 59010 carries analogous coding limitations, and we lacked data on pathogen type or antibiotic timing—potential unmeasured confounders. The deliberate exclusion of patients with renal medullary necrosis was necessary to preserve cohort homogeneity from a pathophysiological standpoint, as medullary necrosis represents an ischemic process that triggers a “necroinflammation” autoamplification loop, potentially obscuring early warning signals and confounding prediction in mixed cohorts. However, this exclusion strategy, which relied on the final discharge ICD-9 code, inevitably excludes those severe cases that progressed from non-necrosis to necrosis during their hospitalization, potentially underestimating the full spectrum of disease severity. Consequently, our model may underestimate the full spectrum of disease progression and its applicability to the most critically ill subgroups, such as those evolving into septic shock, remains limited.

This selection bias should be carefully weighed when interpreting our findings, and external validation in cohorts with higher disease severity is urgently needed to define the model’s true upper limit of performance. Future work should prioritize external validation in geographically diverse cohorts to confirm generalizability. Integration of this model into electronic health records with automated real-time calculation would facilitate seamless clinical adoption. Longitudinal time-series data should be incorporated to generate dynamic risk scores that update with each new measurement, potentially improving predictive performance. Machine learning approaches, including gradient boosting and neural networks, may reveal nonlinear interactions among predictors. Further refinement could involve combining our model with inexpensive inflammatory markers (e.g., C-reactive protein, procalcitonin) to boost sensitivity. Subgroup analyses across age, sex, and comorbidity strata would help identify populations that benefit most. Cost-effectiveness studies are warranted to evaluate whether model-guided early intervention reduces overall healthcare expenditures. Ultimately, prospective cluster-randomized interventional trials are needed to determine whether model-guided decision-making translates into measurable improvements in patient outcomes, including reduced sepsis incidence and mortality.

## 5 Conclusion

This study successfully developed and internally validated a clinical prediction model that quantifies, at hospital admission, the risk of a subsequent sepsis diagnosis in acute pyelonephritis patients without renal medullary necrosis. In conclusion, we developed and internally validated a concise, five-variable risk prediction model (Charlson Index, bicarbonate, calcium, MCV, and admission age) that identifies acute pyelonephritis patients without renal medullary necrosis who are at elevated risk of progressing to sepsis. The model demonstrates good discriminative performance (Development AUC = 0.820; Validation AUC = 0.830) and satisfactory calibration, with decision curve analysis confirming its net clinical benefit across clinically relevant threshold ranges (development: 1–80%; validation: 1–76%). By leveraging routinely available admission parameters and rigorous statistical methodology, the model offers clinicians a practical, bedside quantitative aid for instantaneous risk stratification at hospital admission, enabling early clinical vigilance and prioritized monitoring. Future work should prioritize prospective, multicenter external validation and explore the integration of this model into electronic health record systems for dynamic, automated early warning.

## Data Availability

The original contributions presented in the study are included in the article/Supplementary material, further inquiries can be directed to the corresponding authors.
